# Single Synapse LTP: A Matter of Context?

**DOI:** 10.3389/fncel.2019.00496

**Published:** 2019-11-12

**Authors:** Dennis L. H. Kruijssen, Corette J. Wierenga

**Affiliations:** Department of Biology, Science for Life, Utrecht University, Utrecht, Netherlands

**Keywords:** synaptic plasticity, long-term potentiation, dendritic spine, glutamate uncaging, molecular pathways, synaptic crosstalk

## Abstract

The most commonly studied form of synaptic plasticity is long-term potentiation (LTP). Over the last 15 years, it has been possible to induce structural and functional LTP in dendritic spines using two-photon glutamate uncaging, allowing for studying the signaling mechanisms of LTP with single synapse resolution. In this review, we compare different stimulation methods to induce single synapse LTP and discuss how LTP is expressed. We summarize the underlying signaling mechanisms that have been studied with high spatiotemporal resolution. Finally, we discuss how LTP in a single synapse can be affected by excitatory and inhibitory synapses nearby. We argue that single synapse LTP is highly dependent on context: the choice of induction method, the history of the dendritic spine and the dendritic vicinity crucially affect signaling pathways and expression of single synapse LTP.

## Introduction

Synaptic plasticity is the fundamental cellular correlate of learning. By the strengthening and weakening of specific connections, information processing in the brain is changed and memories are formed. The most studied form of plasticity is long-term potentiation (LTP). As first identified in the rabbit brain by Bliss and Lømo (1973), repeatedly stimulating synapses can lead to long lasting enhancement of synaptic strength. This phenomenon has been extensively studied and characterized in a variety of brain regions and species. The majority of studies use electrical stimulation of axon bundles to induce and measure LTP in brain slices. LTP can also be induced pharmacologically by applying for example an N-methyl-d-aspartate (NMDA) receptor agonist. These approaches induce LTP in bulk: many synapses on dendritic branches of multiple neurons are potentiated at the same time. Electrophysiological recordings and biochemical analysis of the underlying signaling pathways have provided significant insights into the mechanisms of LTP (Malenka and Bear, 2004; Citri and Malenka, 2008; Sjöström et al., 2008; Mayford et al., 2012; Bliss and Collingridge, 2013; Herring and Nicoll, 2016; Nicoll, 2017; Diering and Huganir, 2018). However, this way of inducing LTP does not reflect the physiological situation very well. Under physiological conditions, synaptic inputs are usually not synchronously active in such large numbers, and synaptic plasticity presumably takes place at the scale of individual or small groups of synapses.

The development of two-photon glutamate uncaging almost 20 years ago (Matsuzaki et al., 2001, 2004; Ellis-Davies, 2019) made it possible to activate and potentiate individual synapses. Using a caged compound of the main excitatory neurotransmitter, individual excitatory synapses on spines can be activated with focused laser light at a near-physiological spatial and temporal scale (Matsuzaki et al., 2001) and plasticity can be induced by repetitive stimulation (Matsuzaki et al., 2004). Since then, many studies have used two-photon glutamate uncaging to study the induction, expression and signaling pathways of LTP in single synapses. These studies have significantly improved our understanding of the mechanisms underlying LTP at the single synapse level. However, differences and disagreements between studies also reveal the limitations of our current understanding of single synapse LTP.

The goal of this review is to summarize and compare studies that used two-photon glutamate uncaging to gain insight into single synapse LTP signaling pathways. We will compare different methods to induce LTP in single synapses and discuss how the choice of LTP induction protocol may affect LTP expression and signaling pathways. We will summarize the signaling pathways that are triggered in a single spine during LTP induction using two-photon uncaging and discuss the possibility that multiple LTP pathways may exist, which can be differentially activated depending on the experimental conditions. Finally, we discuss how LTP at a single synapse can affect plasticity at other excitatory and inhibitory synapses on the same dendrite, suggesting that potentiation of an individual synapse should always be considered in the context of its direct dendritic vicinity.

## Induction of Single Synapse LTP

Two-photon microscopy (Denk et al., 1990; Masters and So, 2004) utilizes the physical principle of two-photon excitation: fluorescent proteins are excited only in a femtoliter-sized volume inside the laser beam focus, where the laser light intensity is high enough for excitation by two coincident photons (Zipfel et al., 2003; Svoboda and Yasuda, 2006). Individual long wavelength photons have low energy, which means that out-of-focus laser light causes minimal photodamage. In addition, long wavelength light can penetrate deep into tissue without scattering, allowing live two-photon imaging of small structures, such as dendritic spines up to 1 mm deep into living brain tissue, to be performed (Denk and Svoboda, 1997; Helmchen and Denk, 2005). With the same precision, the two-photon principle allows for precise photolysis of “caged compounds” – biologically active molecules that are inert until exposed to the right wavelength of light (Soeller and Cannell, 1999). The development of MNI-glutamate, a caged compound of the main excitatory neurotransmitter which has a high two-photon cross section, allowed stimulation of single excitatory synapses (Matsuzaki et al., 2001) and induction of plasticity at individual spines (Matsuzaki et al., 2004). The development of several Förster Resonance Energy Transfer (FRET) probes that can detect the activity of signaling molecules on the level of the single spine allowed studying the underlying pathways of LTP with greater detail than ever before (Yasuda, 2012; Ueda et al., 2013; Nakahata and Yasuda, 2018). With these technological advancements, it is now possible to elucidate the mechanisms that are involved in LTP on the level of single excitatory synapses.

The first study to report single synapse LTP was performed by Matsuzaki et al. (2004). Upon performing repeated glutamate uncaging on single dendritic spines, the stimulated spines rapidly grew and remained enlarged for up to 100 min, while unstimulated spines on the same dendrites were unaffected. The authors furthermore showed that spine growth crucially depended on NMDA receptor activation and was similar to spine growth after electrical stimulation. Spine growth was accompanied by a corresponding increase in A-Amino-3-Hydroxy-5-Methyl-4-Isoxazolepropionic Acid (AMPA) receptor-mediated postsynaptic currents, linking growth of the spine head with functional plasticity of the excitatory synapse. Since the pioneering work by Matsuzaki and colleagues, the two-photon glutamate uncaging technique was quickly adopted by the LTP field, and multiple labs have performed single synapse LTP experiments since then. A major benefit of using glutamate uncaging to study LTP is the high spatial and temporal precision of the stimulus. As presynaptic stimulation is no longer required, it allows for isolating the postsynaptic component of LTP.

Single synapse LTP is generally induced by repeated uncaging pulses. The repeated activation of postsynaptic glutamate receptors results in calcium influx, most prominently via NMDA receptors, which triggers plasticity at the stimulated spine. Induction protocols for LTP differ in several aspects, which may significantly influence downstream signaling and LTP expression. The number of uncaging pulses typically ranges from 30 to 60, and the stimulation frequency usually lies between 0.5 and 2 Hz. Both these parameters will likely affect the total amount of calcium entering the postsynaptic cell and the level of activation of downstream calcium sensing proteins (Fujii et al., 2013). The duration of a single uncaging pulse typically lies between 0.5 and 6 ms. The pulse duration determines the time receptors are exposed to glutamate as well as the total amount of glutamate that is uncaged, affecting the duration and level of activation of glutamate receptors (AMPA receptors and NMDA receptors) in the postsynapse. The uncaging beam is typically aimed 0.5 μm from the spine head to prevent photodamage to the spine. The distance between the location of glutamate release and the spine will impact the diffusion time of glutamate to the receptors. While glutamate uncaging is highly local, especially during strong stimulation glutamate spillover to extrasynaptic receptors and presynaptic receptors (such as metabotropic glutamate receptors) is likely to occur (Rusakov and Kullmann, 1998; Chalifoux and Carter, 2011).

**FIGURE 1:**
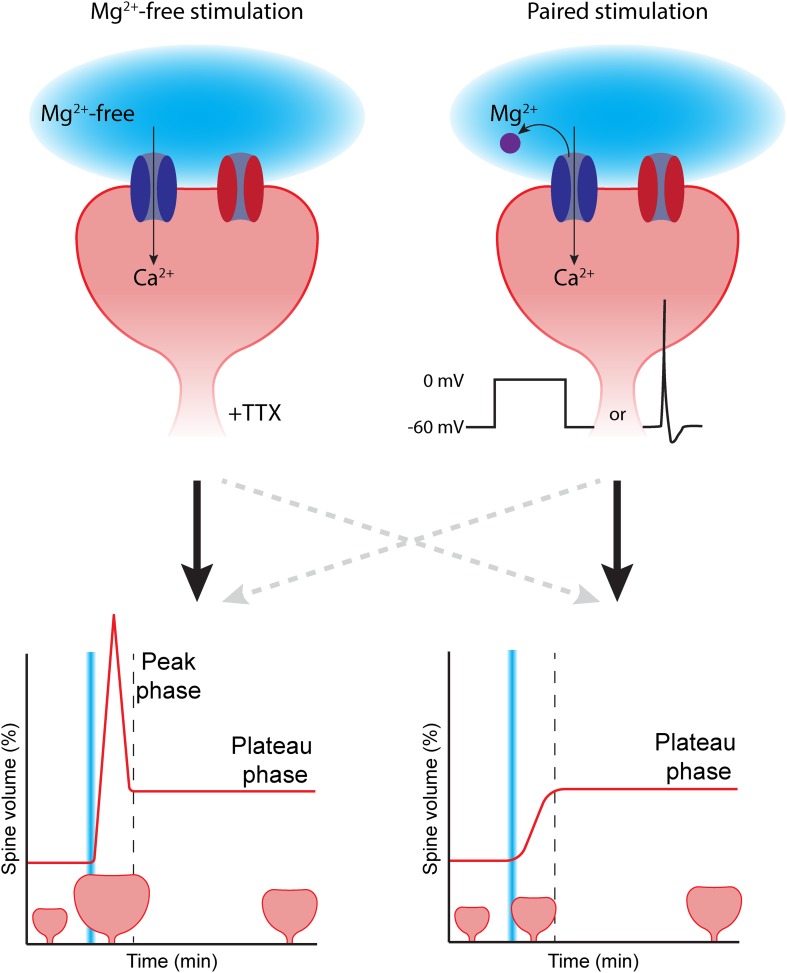
The choice of LTP induction method can affect spine growth. To induce spine growth and functional LTP in single synapses, activation of NMDA receptors (dark blue) is required. Removing the magnesium block (purple) from the NMDA channel pore can be achieved in two ways: **(Left)** glutamate uncaging (light blue) is performed in the absence of extracellular magnesium (Mg^2+^-free). In this case, tetrodotoxin (TTX) is added to prevent aberrant plasticity due to spontaneous activity. This type of stimulation typically induces rapid, strong initial growth (peak phase), after which the spine volume stabilizes at a lower level (plateau phase). **(Right)** In paired protocols, two-photon glutamate uncaging (light blue) is paired with depolarization (in voltage clamp by increasing the holding potential, or in current clamp by inducing a backpropagating action potential). Paired stimulation typically leads to a gradual growth of the dendritic spine over time. AMPA receptors in the spine head are depicted in red. The dashed gray lines reflect that the correlation between stimulation protocol and temporal profile of spine growth is not absolute.

NMDA receptor activation is one of the crucial events for LTP to occur, and different methods are used to ensure NMDA receptor activation during glutamate uncaging at spines (Figure 1). Here, we roughly divide these protocols into two categories. The first category is based on the protocol by Matsuzaki and colleagues. To achieve NMDA receptor activation, glutamate uncaging is performed in absence of extracellular magnesium ions to remove blockage of the channel pore (Tanaka et al., 2008; Lee et al., 2009; Patterson et al., 2010; Tønnesen et al., 2014; Oh et al., 2015; Harward et al., 2016). Caged compounds are known to exhibit antagonist activity at gamma-aminobutyric acid (GABA)_*A*_ receptors (Fino et al., 2009; Matsuzaki et al., 2010; Ellis-Davies, 2019). Therefore, tetrodotoxin (TTX, a sodium channel blocker) is usually added to the bath solution under magnesium-free conditions to prevent epileptiform-like activity and unwanted plasticity. The second category of protocols pairs glutamate uncaging with postsynaptic depolarization or postsynaptic action potentials to relieve the magnesium block from the NMDA receptors. This type of protocol typically requires electrical access to the postsynaptic cell via a patch clamp electrode. In voltage clamp experiments, the cell is depolarized (typically to 0 mV) while glutamate is uncaged at a spine (Matsuzaki et al., 2004; Harvey and Svoboda, 2007; Lee et al., 2009). In current clamp experiments, current is injected to induce action potential firing while glutamate is uncaged at a spine (Tanaka et al., 2008; Hayama et al., 2013). Alternatively, all-optical uncaging LTP experiments can be performed by pairing optogenetically induced postsynaptic depolarization with glutamate uncaging (Zhang et al., 2008).

The majority of studies have used magnesium-free protocols, which has the great advantage that electrical access to the postsynaptic cell is not required and the studied neuron can be left unperturbed. However, performing experiments in magnesium-free extracellular solution is far from physiological: NMDA receptors are constantly “primed” for activation and addition of TTX is required to block all spontaneous electrical activity. Furthermore, the absence of magnesium could affect several other cellular processes that require magnesium (de Baaij et al., 2015). Paired protocols mimic physiological conditions more accurately. Under physiological conditions, the magnesium block will be relieved by depolarization of the postsynaptic membrane (Gambino et al., 2014). An additional advantage is that the use of patch clamp electrophysiology allows recording of the uncaging-induced excitatory postsynaptic current (uEPSC). This way, the laser power can be tuned to induce uEPSCs with amplitudes that are similar to single synapse EPSCs (typically ∼10–20 pA) to mimic synaptic glutamate levels (Matsuzaki et al., 2001, 2004; Harvey and Svoboda, 2007; Steiner et al., 2008; Lee et al., 2009; Hill and Zito, 2013). However, the use of electrophysiology makes paired protocols more invasive. Signaling molecules that are required for LTP may “wash out” while perfusing the cell with internal solution from the patch pipette, thereby reducing or abolishing the ability to induce LTP (Malinow and Tsien, 1990; Matsuzaki et al., 2004; Tanaka et al., 2008).

In conclusion, the choice of protocol involves several practical and biological considerations, such as the need for patch clamp electrophysiology, washout of signaling molecules, and resemblance of the physiological situation. It is important to realize that these protocols are not completely interchangeable: in the next section, we will discuss how the induction protocol may affect the magnitude and temporal profile of LTP expression.

## Expression of Single Synapse LTP

Inducing LTP in a synapse has two major effects: the number of postsynaptic AMPA receptors is increased and the spine volume is enlarged. After LTP, a presynaptic stimulus will induce a postsynaptic current with larger amplitude than before. This is largely due to an increase of AMPA receptors in the postsynaptic membrane (Kessels and Malinow, 2009; Huganir and Nicoll, 2013; Moretto and Passafaro, 2018). Matsuzaki et al. (2004) showed that also in single potentiated spines, the AMPA receptor-mediated currents increase within minutes after stimulation. Many LTP induction paradigms, such as high-frequency stimulation, theta burst stimulation and optical stimulation of afferents lead to persistent spine growth, which was shown by fluorescence imaging (Lang et al., 2004; Okamoto et al., 2004; De Roo et al., 2008; Wiegert et al., 2018) and electron microscopy (Van Harreveld and Fifkova, 1975; Buchs and Muller, 1996; Bourne and Harris, 2011). *In vivo*, spine volumes fluctuate and spines are continuously formed and removed (Caroni et al., 2012; Berry and Nedivi, 2017). Spine dynamics are enhanced after experience and are thought to support long-lasting changes in neural circuits during experience-dependent plasticity (Holtmaat et al., 2006; Hofer et al., 2009; Roberts et al., 2010). For instance, specific spines grow during a motor learning task, and inducing shrinkage of these spines disrupts the acquired motor skill (Hayashi-Takagi et al., 2015). Spine growth is largely attributed to remodeling of actin, which is highly enriched in spines. When a spine is potentiated, polymerization of actin in the spine head leads to more filamentous actin and a bigger spine (Matsuzaki et al., 2004; Okamoto et al., 2004; Harvey et al., 2008; Bosch and Hayashi, 2012; Nakahata and Yasuda, 2018). These morphological changes (actin polymerization and spine growth) and functional changes (increase in AMPA receptors) are often correlated but might be regulated independently.

To monitor the expression of LTP in individual synapses, the increase in ampltidue of the uEPSC can be quantified. The uEPSC at a spine can go up 40–120% within minutes after LTP induction (Matsuzaki et al., 2004; Harvey and Svoboda, 2007; Steiner et al., 2008; Tønnesen et al., 2014). While quantifying uEPSC increase is a useful method to assess functional LTP, it can be technically challenging. Other than on the strength of the synapse, the uEPSC amplitude also depends on the laser power at the uncaging location, the local caged glutamate concentration, and the distance of the uncaging spot to the postsynaptic density, all of which are challenging to keep stable at growing spines during the experiment. Furthermore, electrical access to the postsynaptic cell is required. Quantification of the morphological changes of the spine head is therefore often used as an alternative measure.

Spine growth can be quantified using two-photon microscopy images of the stimulated spine over time. Depending on the initial size and the protocol used, spine heads show (transient) growth up to 200–400% (Matsuzaki et al., 2004; Bosch et al., 2014; Tønnesen et al., 2014; Murakoshi et al., 2017). Spine size correlates strongly with synapse strength under resting conditions *in vitro* (Matsuzaki et al., 2001; Noguchi et al., 2005; Zito et al., 2009) and *in vivo* (Noguchi et al., 2011). Because of this strong correlation, as well as the technical challenges of quantifying uEPSC amplitude over time, spine growth is often taken as a proxy for functional LTP. The correlation between size and function is, however, not absolute: morphological and functional changes might not match perfectly in the first hour after LTP induction (Bosch et al., 2014; Meyer et al., 2014), and functional LTP can also occur in the absence of spine head growth (Araya et al., 2014). It is important to mention that the high laser power used for glutamate uncaging can induce photodamage and swelling of the spine head when the laser beam is aimed too close to the spine head. Swelling due to photodamage could potentially confound actual spine growth due to LTP but can be prevented by aiming the laser beam ∼0.5–1 μm away from the spine head.

### Other Morphological Changes

The increase in spine head size is not the only morphological change upon LTP. Several studies have reported shorter and/or thicker spine necks after LTP induction (Tanaka et al., 2008; Araya et al., 2014; Bosch et al., 2014; Tønnesen et al., 2014). These changes in spine neck geometry seem to be consistent with an increase in electrical coupling (Araya et al., 2006, 2014; Tønnesen et al., 2014) and may provide a mechanism for synaptic strengthening independent of AMPA receptor regulation. In addition, glutamate uncaging may induce remodeling of the extracellular space, possibly via glial responses (Tønnesen et al., 2018).

Glutamate uncaging bypasses the need of activating glutamate release of the presynaptic terminal and allows isolation of the postsynaptic component of LTP. However, the presynaptic bouton is probably also affected by glutamate uncaging. After a putative LTP-inducing uncaging protocol, boutons increase their size by ∼50% gradually over the course of 1–3 h, maintaining the correlation between bouton size and spine size (Meyer et al., 2014).

It has also been reported that repeated glutamate uncaging on the dendrite can induce the formation of a new dendritic spine at the uncaging location within seconds, which can become functional within 30 min (Kwon and Sabatini, 2011; Hamilton et al., 2012). In a different study it was shown that new spines rapidly mature and become functional (Zito et al., 2009). New spines have the capacity to grow upon glutamate uncaging, which significantly increases their persistence (Hill and Zito, 2013).

### Variability in Spine Growth

There is a remarkable level of variability in the reported time course and magnitude of spine growth between studies, even within the same brain region and cell type (Tanaka et al., 2008; Bosch et al., 2014). Many studies report an initial peak (or transient phase) of a few minutes in which the spine grows drastically. This peak growth can range from 100 to 400%. This phase is then followed by a plateau (or sustained phase) where the spine growth declines and stabilizes, typically at 50–100% (Harvey and Svoboda, 2007; Tanaka et al., 2008; Patterson et al., 2010; Bosch et al., 2014; Tønnesen et al., 2014; Oh et al., 2015; Harward et al., 2016). Other studies report a gradual spine growth over the course of 5–10 min, which then stabilizes at a plateau, without a significant peak (Harvey and Svoboda, 2007; Tanaka et al., 2008; Hayama et al., 2013; Hu et al., 2019). Even when comparing studies that show a similar temporal pattern of spine growth, peak and plateau magnitudes often vary significantly. One could wonder to what extent extreme peak spine growth resembles the physiological situation.

Technical differences such as differences in quantification methods and model systems could partially explain this remarkable variability, but other factors may be more vital. First of all, the initial size of the spine before induction of LTP matters: small spines have a larger growing capacity than spines that are already larger to begin with (Matsuzaki et al., 2004; Tanaka et al., 2008). It has even been suggested that the large spines cannot grow upon stimulation at all (Matsuzaki et al., 2004; Tanaka et al., 2008). Although it is difficult to compare initial spine size between studies, a difference in initial spine size may explain some of the observed differences in spine growth magnitude.

More importantly, the choice of LTP induction protocol will crucially affect the magnitude and time course of spine growth. This was first observed by Tanaka et al. (2008). When they paired glutamate uncaging with backpropagating action potentials, it led to a gradual growth of the spine, reaching close to 150% growth. However, when they performed glutamate uncaging in absence of extracellular magnesium, spine growth showed an initial peak in which spine volume reached twofold growth (100%), after which spine growth declined to reach a plateau phase at 50% (Tanaka et al., 2008). Similarly, Harvey and Svoboda (2007) reported a gradual spine growth of 80% when using a paired protocol. A similar magnesium-free protocol resulted in 175% peak growth, declining to a plateau at 75% growth (Harvey and Svoboda, 2007). These studies clearly suggest that different induction protocols activate different intracellular signaling pathways, resulting in differences in spine growth. Typically, magnesium-free induction protocols lead to peak-plateau growth, while paired protocols often induce gradual spine growth (Figure 1; although this correlation is not absolute, Matsuzaki et al., 2004; Zhang et al., 2008; Lee et al., 2009).

Why do different induction protocols lead to such remarkable differences in spine growth? The choice of induction protocol likely affects how downstream signaling pathways are activated. This already occurs at the level of calcium concentration elevation, the key signal for LTP. While calcium influx is typically restricted to the spine head in magnesium-free stimulation protocols, paired protocols also cause an increase of calcium concentration in the dendritic shaft (see below). This differential spatial calcium profile may also lead to differential activation of downstream signaling molecules, and it is interesting to speculate how these could be linked to the peak and plateau phases of spine growth. For instance, the study by Tanaka et al. (2008) showed that the paired protocol involved BDNF signaling and protein synthesis to induce spine growth, while spine growth was independent of BDNF in the magnesium-free protocol. However, a more recent study observed that BDNF also affects spine growth after a magnesium-free protocol (Harward et al., 2016). These data suggest that there is not a single universal mechanism for the expression of LTP in spines. Multiple modes of LTP may exist, and different protocols may activate different signaling mechanisms. We will discuss these signaling pathways in the next sections. We will first describe which pathways are activated when LTP is induced in single spines, followed by a discussion on how signaling pathways between nearby synapses can interact.

## Single Synapse LTP Signaling Pathways

In this section, we discuss the signaling pathways that are activated when a single spine is potentiated. Expression of LTP has been extensively examined using chemical or electrical LTP induction, in which multiple synapses are activated in many neurons simultaneously and signaling pathways are triggered in a large part of the neuron. These studies have established that calcium influx through NMDA receptors and subsequent activation of CaMKII are essential for LTP. Downstream signaling pathways eventually lead to actin remodeling and the insertion of AMPA receptors, resulting in a stronger synapse. Here we limit our discussion to studies using two-photon glutamate uncaging to induce LTP in a single synapse. By inducing LTP in a single synapse, it is possible to study the activation of molecules in LTP signaling pathways with the highest temporal and spatial detail.

### Glutamate Receptors

Glutamate uncaging on a dendritic spine activates AMPA receptors and NMDA receptors in the postsynaptic density (although glutamate receptors can also be found extrasynaptically and presynaptically, Parsons and Raymond, 2014; Bouvier et al., 2018). AMPA receptors mainly conduct sodium and potassium ions and are largely responsible for synaptic membrane depolarization in the spine. Binding of glutamate to NMDA receptors is usually not sufficient to open the channel, as they are blocked by magnesium. Only when the postsynaptic membrane is sufficiently depolarized, during AMPA receptor activation, a backpropagating action potential or a dendritic spike, the magnesium block is relieved and NMDA channels open. When NMDA receptors are activated, it leads to the rapid influx of calcium ions through the channel pore into the dendritic spine. Many studies have demonstrated that NMDA receptor activation is required for the growth of single spines (Matsuzaki et al., 2004; Harvey and Svoboda, 2007; Zhai et al., 2013; Tang and Yasuda, 2017).

Not all spines contain both AMPA receptors and NMDA receptors. AMPA receptor content is correlated to spine size, and the smallest spines can be silent, meaning that they contain no AMPA receptors and therefore no current can be measured when the spine is exposed to glutamate. These silent spines however do contain NMDA receptors (Béïque et al., 2006; Busetto et al., 2008). This allows these spines to undergo LTP by growing and recruiting AMPA receptors.

Besides ionotropic glutamate receptors, dendritic spines also contain group I metabotropic glutamate receptors (mGluRs). These mGluRs are enriched immediately next to the postsynaptic density (Scheefhals and MacGillavry, 2018). When glutamate uncaging is performed at a dendritic spine, it is likely that mGluRs will also be activated, especially when long uncaging pulses or many repetitions are used. When the metabotropic glutamate receptors are blocked during the induction of single synapse LTP, spine growth typically remains intact (Matsuzaki et al., 2004; Zhai et al., 2013; Bosch et al., 2014; Colgan et al., 2018), suggesting they do not play a major role in LTP induction.

### Calcium

Calcium entering the spine via NMDA receptor activation is considered the key signal to trigger LTP. During a single synapse LTP induction protocol, each uncaging stimulus leads to a brief influx of calcium into the dendritic spine (Lee et al., 2009; Zhai et al., 2013; Colgan et al., 2018). There is a tight inverse correlation between spine head volume and calcium levels: uncaging on a smaller spine leads to a higher calcium concentration (Noguchi et al., 2005; Sobczyk et al., 2005). This can partly be explained by geometric differences, but different subunit composition of NMDA receptors in smaller spines may also play a role (Sobczyk et al., 2005). Depending on the geometry of the spine neck (length and width), some calcium will diffuse from the spine head into the dendritic shaft (Noguchi et al., 2005; Zhai et al., 2013).

While calcium influx through NMDA receptors is crucial for LTP induction, other sources of calcium can be involved as well. When glutamate uncaging is paired with postsynaptic depolarization, voltage-gated calcium channels (VGCCs) in the dendrite and spine get activated (Lee et al., 2009; Müllner et al., 2015) and this will lead to additional calcium influx. An experiment by Zhai et al. (2013) suggests that VGCCs do not play a role in the induction of LTP under magnesium-free conditions, but may affect the plateau level of spine growth.

### Calcium-Sensing Proteins

The increase of calcium concentration upon NMDA receptor activation is sensed by Calcium/calmodulin kinase II (CaMKII), and activation of CaMKII is essential for the induction of LTP. CaMKII can associate with several structures in the spine head, such as filamentous actin and several proteins in the postsynaptic density (Okamoto et al., 2004; Hell, 2014; Kim et al., 2015). Changes in local CaMKII levels may occur after single synapse LTP induction. CaMKII concentration in the spine has been reported to temporarily drop for 5 min (Bosch et al., 2014), or to slightly but persistently increase after LTP induction (Zhang et al., 2008). As changes in the concentration of CaMKII are also dependent on changes in spine volume, it is important to mention that these studies use different induction protocols (magnesium-free versus paired) and observe a different temporal pattern and amplitude of spine growth. Both studies agree that the total amount of bound (as opposed to freely diffusing) CaMKII in the spine head increases after LTP induction (Zhang et al., 2008; Bosch et al., 2014). It was previously shown that the amount of bound CaMKII in the spine correlates strongly with spine size and uEPSC amplitude under baseline conditions (Asrican et al., 2007), suggesting that the trapping of CaMKII in the spine head is directly related to strengthening of the spine during LTP. On longer timescales, the fraction of bound CaMKII returns to baseline (Asrican et al., 2007; Zhang et al., 2008), indicating that unbound CaMKII slowly diffuses to the spine to restore the ratio of bound/unbound CaMKII.

CaMKII is activated by calcium and the calcium-binding protein calmodulin. Calmodulin associates with and dissociates from CaMKII within seconds. The association of calmodulin and CaMKII does not accumulate during a single synapse LTP induction protocol (Chang et al., 2019). CaMKII activation however does increase with every uncaging pulse, thereby integrating multiple calcium signals. CaMKII even stays active for up to 1 min after the end of the induction protocol (Lee et al., 2009; Chang et al., 2017, 2019). This accumulation and persistence of the signal can be explained by autophosphorylation (at the threonine 286 residue), allowing CaMKII to remain active after calcium/calmodulin unbinds. Autophosphorylation of CaMKII is important for LTP induction: the slower inactivation rate permits signal integration at relatively low frequency stimulation. Only at extremely high frequencies (>8 Hz) can repeated stimulation sustain CaMKII activation without autophosphorylation (Chang et al., 2017).

CaMKII plays an important role in spine growth. Multiple studies show that pharmacological inhibition or genetic knockout of CaMKII strongly reduces the plateau phase of spine growth, while peak growth is maintained (Matsuzaki et al., 2004; Lee et al., 2009; Murakoshi et al., 2011; Hedrick et al., 2016; Incontro et al., 2018; Saneyoshi et al., 2019). Using a photoactivatable CaMKII inhibitor, Murakoshi et al. (2017) demonstrated that CaMKII activation is required for only 1 min during LTP induction. Interestingly, both the peak and plateau of spine growth were strongly reduced when the inhibitor was activated during the entire LTP induction protocol. When the inhibitor was activated 30 s after the start of the induction protocol, only plateau growth was reduced while peak growth remained (Murakoshi et al., 2017). These data suggest that the peak and plateau growth require different durations of CaMKII activation but are in disagreement with experiments using pharmacological inhibition of CaMKII (discussed above).

The spatial extent of CaMKII activation depends on the LTP induction protocol. In a typical magnesium-free induction protocol, CaMKII activation is mostly restricted to the spine head (Lee et al., 2009), although a small amount of active CaMKII might be found in the dendritic shaft (Chang et al., 2017). However, when glutamate uncaging is paired with postsynaptic depolarization, dendritic VGCCs are activated and as a result CaMKII is also strongly activated in the dendritic shaft (Lee et al., 2009).

In addition to CaMKII, the phosphatase calcineurin (CaN) is also activated in the spine head and dendritic shaft when calcium levels increase. While CaMKII is sensitive to both the frequency and number of uncaging stimuli, CaN is less sensitive to stimulation frequency and mainly responds to the number of stimuli (Fujii et al., 2013). Calcineurin activity is typically associated with spine shrinkage and synaptic depression (Zhou et al., 2004; Hayama et al., 2013; Nabavi et al., 2013; Oh et al., 2015).

### GTPases: Ras, RhoA, Cdc42, Rac1

During and after LTP induction, several small GTPases are activated in the dendritic spine via both CaMKII-dependent and -independent pathways. Small GTPases are enzymes that often function as “molecular switches” in biological signaling pathways and play an important role in regulating the synaptic actin cytoskeleton and plasticity (Hotulainen and Hoogenraad, 2010; Patterson and Yasuda, 2011). Harvey et al. (2008) used a FRET-sensor to show that the small GTPase Ras is activated in the dendritic spine within 1 min after glutamate uncaging. Activity decays substantially in 5 min, but some Ras stays activated for at least 15 min. Ras activation is partly dependent on CaMKII (Harvey et al., 2008), likely through phosphorylation of the Ras GTPase activating protein SynGAP (Araki et al., 2015), but Ras activation also depends on PI3K and PKC activity (Harvey et al., 2008). Ras presumably acts via the extracellular signal-regulated kinase ERK via the Ras-MEK pathway. ERK activation in the spine peaks within 5 min after LTP induction and lasts for 20 min (Tang and Yasuda, 2017). Ras-ERK signaling plays an important role in spine growth: interfering with Ras activation or with its downstream Raf-MEK-ERK pathway reduces the magnitude of the plateau, but not of the peak spine growth (Harvey et al., 2008; Zhai et al., 2013). When both CaMKII and the Ras-Raf-MEK-ERK pathway are inhibited, plateau spine growth is almost completely abolished, suggesting that these pathways together are responsible for the majority of spine growth in the plateau phase (Harvey et al., 2008).

RhoA, a member of the Rho subfamily of GTPases, is also activated in the stimulated spine within 30 s upon LTP induction. While the level of activity largely decays within 5 min, some activity remains for 30 min. Another Rho GTPase family member, Cdc42, shows similar activation kinetics. RhoA and Cdc42 activation is partially dependent on CaMKII signaling. Functional LTP is completely abolished when RhoA or Cdc42 are inhibited. Inhibition of RhoA or its downstream effector Rock reduces both the peak phase and the plateau phase of spine growth, while interfering with Cdc42 or its downstream effector Pak affects plateau phase spine growth only (Murakoshi et al., 2011). Experiments by Hedrick et al. (2016) suggest that Cdc42 activation can be downstream from autocrine BDNF signaling (see below), while RhoA is activated independently.

A third Rho GTPase family member Rac1 is also activated rapidly in the dendritic spine upon LTP induction, partly in a CaMKII- and BDNF-dependent manner. Rac1 shows stronger sustained activation than RhoA and Cdc42. Interfering with Rac1 signaling significantly reduces both the peak phase and plateau phase of spine growth (Hedrick et al., 2016). Recently, it was shown that sustained activation of Rac1 is regulated by the guanine nucleotide exchange factor Tiam1. Tiam1 forms a complex with activated CaMKII, and both proteins reciprocally keep each other active. Interfering with Tiam1 or the complex formation between CaMKII and Tiam1 significantly affects spine growth (Saneyoshi et al., 2019).

Together, the picture emerges that glutamate uncaging induces spine growth and functional LTP via multiple, and partially overlapping, GTPase pathways (Nakahata and Yasuda, 2018).

### PKC and PKA Signaling

Classical protein kinase C (PKC) family proteins are typically activated in the presence of calcium and the lipid diacylglycerol (DAG) (Lipp and Reither, 2011). It has been shown that specifically PKCα mediates the plateau phase of spine growth (Colgan et al., 2018). PKC activation occurs in the dendritic spine and is extremely rapid: PKC is activated after every uncaging pulse, but activity has already decayed by the time of the next uncaging pulse (at 0.5 Hz). Blocking calcium influx through NMDA receptors completely abolishes PKC activation, and PKC activation and spine growth are reduced when the production of DAG by Phospholipase C (PLC) is inhibited. During LTP induction, PLC is activated by autocrine BDNF-TrkB signaling (see below) and not by mGluR activation (Colgan et al., 2018).

Another important kinase, protein kinase A (PKA), seems to play a modulatory role in LTP (Esteban et al., 2003; Blitzer et al., 1998; Man et al., 2007). PKA activity depends on cyclic AMP levels and is downstream of a variety of G-protein coupled receptors. Single synapse LTP induction leads to rapid activation of PKA in the spine, which decays back to baseline in 5 min. Interestingly, PKA activation was found to be downstream of NMDA receptor activation (Tang and Yasuda, 2017). LTP does not require PKA activation, but PKA activation can boost single synapse LTP (Govindarajan et al., 2011; Yagishita et al., 2014). However, PKA activation originating from a single stimulated spine may not be sufficient for this boosting effect, and more global PKA activation, for instance via dopaminergic neuromodulatory signals (Yagishita et al., 2014), may be required.

### Actin

Actin is the major structural component of the dendritic spine, and spine growth requires actin remodeling. Matsuzaki et al. (2004) already showed that single spine growth is prevented in the presence of Latrunculin A, a drug that sequesters actin monomers and prevents actin polymerization. In resting conditions, two pools of actin can be found in the dendritic spine: a highly dynamic pool located at the tip of the spine head and a very stable pool at the base of the spine. After LTP induction, a third “enlargement” pool appears, and this pool seems to be responsible for spine growth (Honkura et al., 2008).

Upon LTP induction, the amount of actin in the spine and several actin-interacting proteins (Arp2/3, profilin, Aip1, drebrin, α-actinin, cofilin) increases in parallel with spine growth (Bosch et al., 2014). Some of these proteins (Arp2/3, Aip1, actin, cofilin) increase rapidly during peak growth, and the concentration of cofilin in the spine head remains elevated for at least 30 min. Upon LTP induction, cofilin is phosphorylated by LIM kinase, which is downstream of the Cdc42-Pak and RhoA-Rock pathways discussed above (Bosch et al., 2014). Phosphorylation of cofilin is required for the peak and plateau phases of spine growth (Noguchi et al., 2016). In the first few minutes, phosphorylated cofilin presumably severs actin filaments and thereby boosts the nucleation of new actin filaments and branching by Arp2/3, resulting in spine growth. After this initial phase, cofilin is dephosphorylated again and can decorate actin filaments, thereby stabilizing them. In absence of cofilin, the plateau phase of spine growth is abolished (Bosch et al., 2014).

Interestingly, during baseline conditions CaMKII associates with actin filaments in the spine head. When calcium flows into the spine head and activates CaMKII, autophosphorylation of CaMKII causes it to dissociate from filamentous actin, allowing binding of cofilin and other actin regulators to remodel the actin cytoskeleton. After dephosphorylation, CaMKII quickly binds and thereby stabilizes actin filaments. It has been suggested that the rapid and transient (∼1 min time window) dissociation of CaMKII from filamentous actin allows the rapid and transient peak spine growth observed in some studies (Kim et al., 2015). Preventing CaMKII F-actin dissociation strongly reduces functional LTP in slices and strongly reduces fear learning *in vivo* (Kim et al., 2015, 2019).

### AMPA Receptors and Postsynaptic Density

Within minutes after single synapse LTP induction, synaptic strengthening is expressed as an increase in the amount of AMPA receptors on the spine surface (Makino and Malinow, 2009; Patterson et al., 2010; Bosch et al., 2014; Chiu et al., 2017; Soares et al., 2017) and can be measured by an increase in AMPA receptor-mediated currents (Matsuzaki et al., 2004; Harvey and Svoboda, 2007; Steiner et al., 2008; Tønnesen et al., 2014). The increase of AMPA receptors in the postsynaptic density involves receptor phosphorylation (Boehm et al., 2006) and mainly occurs via lateral diffusion in the membrane, but exocytosis of AMPA receptor-containing vesicles also contributes (Makino and Malinow, 2009; Patterson et al., 2010; Chiu et al., 2017; Choquet, 2018). A local increase of exocytosis rate occurs during LTP induction, which seems partially dependent on Ras-ERK-mediated, but CaMKII-independent, pathways. CaMKII signaling is likely involved in anchoring of AMPA receptors to spines (Patterson et al., 2010).

The postsynaptic density (PSD) consists of a cluster of proteins close to the postsynaptic membrane. Important PSD proteins such as PSD95, Homer and Shank act as a scaffold to position and anchor ionotropic and metabotropic glutamate receptors (Scheefhals and MacGillavry, 2018). Remodeling of the PSD during LTP is a complex, multi-step process. Under basal conditions, the size of the PSD strongly correlates with the size of the spine head. After LTP induction, the postsynaptic density increases in size, but components arrive in the spine with a delay compared with the rapid AMPA receptor insertion (Steiner et al., 2008; Bosch et al., 2014; Meyer et al., 2014). In some spines, transient spine growth can be observed after glutamate uncaging, which returns to baseline after ∼2 h without any changes to the PSD (Meyer et al., 2014). After successful single synapse LTP, it takes at least 1 h for the correlation between PSD and spine size to restore (Bosch et al., 2014; Meyer et al., 2014).

### Protein Synthesis

Spine growth can occur in the absence of protein synthesis (Harvey and Svoboda, 2007; Harward et al., 2016), but some single synapse LTP induction protocols require synthesis of new proteins. Tanaka et al. (2008) showed that when single synapse LTP is induced in low extracellular magnesium, spine growth is independent of protein synthesis. However, when a similar induction protocol is paired with postsynaptic spiking in physiological levels of magnesium, spine growth is strongly dependent on protein synthesis (Tanaka et al., 2008). A more recent study showed that spine growth induced under magnesium-free conditions actually does require protein synthesis, but only more than 30 min after LTP induction. This study also shows that the gradual recruitment of the postsynaptic scaffolding protein Homer1b was abolished when protein synthesis was inhibited (Bosch et al., 2014). Another study also showed that protein synthesis is involved in the maintenance of enlarged spines after LTP induction. Govindarajan et al. (2011) showed that spine growth returns to baseline after 2 h, but spine growth could be maintained by pharmacological activation of PKA in the entire slice. This maintenance depended on protein synthesis. When glutamate uncaging was paired with PKA activation in the absence of protein synthesis, spine growth was entirely prevented (Govindarajan et al., 2011). These studies illustrate that protein synthesis may be important for spine growth and functional LTP at the single synapse level under certain circumstances, but it is not clear how exactly it is triggered and when it is required.

### Brain-Derived Neurotrophic Factor (BDNF) Signaling

The neurotrophic factor BDNF has been shown to affect single synapse LTP. Tanaka et al. (2008) suggested that BDNF is released after pairing glutamate uncaging with postsynaptic spiking, but not after glutamate uncaging in magnesium-free conditions. However, Harward et al. (2016) observed that a similar uncaging protocol in magnesium-free conditions does lead to rapid release of BDNF from the stimulated spine, and that this is partially dependent on CaMKII activation. BDNF release resulted in rapid and sustained activation of the BDNF receptor TrkB in the stimulated spine, the dendrite and neighboring spines (Harward et al., 2016). BDNF, via TrkB activation, may promote small GTPase and PKC activation (Hedrick et al., 2016; Colgan et al., 2018). In the Tanaka study, LTP was shown to require protein synthesis, while in the Harward study spine growth was independent of protein synthesis. These studies and others (Bosch et al., 2014) suggest that (autocrine) BDNF signaling can facilitate, but is not absolutely required for, single synapse LTP. They also show that subtle differences in stimulation protocol may lead to remarkable differences and illustrate our limited understanding of under which conditions BDNF is released from dendrites and spines.

### Spine Shrinkage

While we focus here on potentiation of spines, glutamate uncaging has also been used to induce shrinkage of spines and depression of synaptic transmission. Low frequency uncaging at a single spine (90 pulses at 0.1 Hz, paired with depolarization) can induce spine shrinkage, which is accompanied by a decrease in uEPSC amplitude. The shrunken spines can undergo LTP and grow again when exposed to an LTP stimulus. There is an interesting difference between small and large spines: while large spines require mGluR and IP3 receptor activation to shrink, the small spines do not (Oh et al., 2013). Spine shrinkage is dependent on non-ionotropic signaling of NMDA receptors, as it can occur without calcium flux through NMDA receptor channels. Surprisingly, a stimulation protocol that normally induces single synapse LTP leads to spine shrinkage when NMDA receptor-dependent calcium flow is inhibited, revealing that NMDA receptors may activate both pathways in parallel (Stein et al., 2015). We refer interested readers to a more elaborate discussion of the molecular mechanisms involved in spine shrinkage and elimination (Stein and Zito, 2018).

### Multiple Parallel Pathways

Studies on the induction of LTP in individual dendritic spines have revealed the temporal and spatial activation patterns of signaling molecules and pathways during LTP induction and expression. Single synapse LTP involves several, partially overlapping, intracellular signaling pathways, and the time course and magnitude of single synapse LTP is critically shaped by the molecular pathways involved. It will be important to gain a better understanding into the stimuli that trigger the different signaling pathways and how multiple pathways interact within single spines and their direct vicinity.

The majority of studies use a magnesium-free protocol to assure NMDA receptor activation during the stimulation protocol, and signaling pathways with this protocol have been described in great detail (Nishiyama and Yasuda, 2015; Nakahata and Yasuda, 2018). Under physiological conditions, glutamate receptor activation coincides with postsynaptic depolarization during LTP induction, which likely affects the spatial and temporal dynamics of signaling molecules in the stimulated spine and adjacent dendrite. Indeed, in a direct comparison, very different patterns of CaMKII activation were observed in magnesium-free and paired protocols (Lee et al., 2009). In addition, the requirements for protein synthesis and the contribution of BDNF signaling were found to be highly protocol-dependent (Tanaka et al., 2008; Govindarajan et al., 2011). This supports the idea that the spatiotemporal activation patterns of downstream signaling pathways are inevitably shaped by the induction protocol. This is important to realize, as experimental conditions are never fully representative of the *in vivo* physiological conditions. To interpret the intricate signaling pathways in the proper context, it is key to improve our understanding of how and when they are evoked at the single synapse level *in vivo*.

## Interactions Between Synapses

In the previous section we discussed the signaling pathways that can be activated when a single synapse undergoes LTP. Dendrites are tightly packed with hundreds of dendritic spines, and neighboring spines may influence each other. Under physiological conditions, single synapse activation may be rare and multiple synapses are receiving inputs simultaneously. It is therefore important to consider how adjacent synapses can influence each other’s plasticity.

### Crosstalk

**FIGURE 2:**
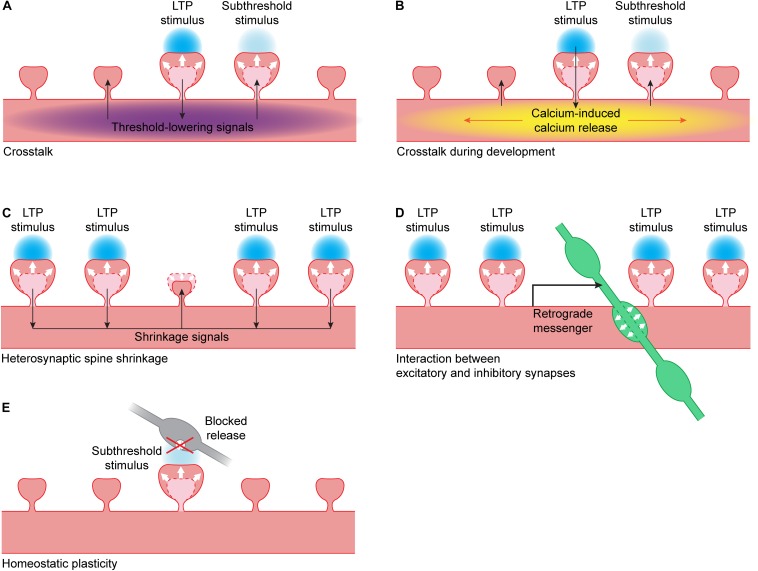
Interactions between synapses. **(A)** When LTP is induced in a single spine using glutamate uncaging (blue), this leads to the spread of threshold-lowering signals (purple) in the dendrite. When a nearby spine receives a stimulus that is normally subthreshold, spine growth will occur. Threshold-lowering signals include the small GTPases Ras, Rac1 and RhoA (Harvey et al., 2008; Murakoshi et al., 2011; Hedrick et al., 2016) and BDNF-TrkB signaling (Harward et al., 2016; Colgan et al., 2018). PKA and ERK activity also spreads over the dendrite but it is unclear if these kinases are able to lower the LTP threshold (Zhai et al., 2013). **(B)** During development, the calcium influx in a single spine during glutamate uncaging can trigger calcium-induced calcium release (yellow). This leads to propagating calcium waves in the dendrite, and a nearby spine receiving a stimulus that is normally subthreshold will now show spine growth (Lee et al., 2016). **(C)** When a cluster of spines undergo LTP, this can lead to the activation of shrinkage signals. These signals can induce shrinkage of an unstimulated dendritic spine nearby (Oh et al., 2015). **(D)** When a cluster of spines undergo LTP, this can lead to the production of a retrograde messenger by the postsynaptic neuron. This messenger can trigger the growth of a presynaptic inhibitory bouton (green) nearby (Hu et al., 2019). **(E)** When vesicle fusion in the presynapse (gray) has been blocked for a prolonged period of time, this can lead to a lowering of the LTP threshold: when a spine receives a stimulus that is normally subthreshold, it will show spine growth (Lee et al., 2010).

Harvey and Svoboda were the first to use glutamate uncaging to show crosstalk can occur between single spines during LTP induction: spines that received a weak (“subthreshold,” 1 ms uncaging pulse) stimulus did not undergo LTP, but they only showed LTP when a nearby spine was stimulated with a strong (4 ms uncaging pulse) LTP-inducing stimulus. It is not clear whether the difference in pulse duration reflects a difference in the level and/or duration of NMDA receptor activation, or a difference in the type of glutamate receptors that are activated. The spine that received the weak stimulus showed the same level of spine growth and functional LTP as the spine that received the strong stimulus (Figure 2A). This crosstalk occurs over a timescale of several minutes and a length scale of 5–10 μm, both in magnesium-free and paired protocols (Harvey and Svoboda, 2007).

Several signaling molecules that are activated during LTP induction can diffuse out of the stimulated spine and affect signaling in neighboring spines. While calcium influx and CaMKII activation are brief and mostly restricted to the dendritic spine (when using a magnesium-free induction protocol) (Harvey et al., 2008; Lee et al., 2009; Otmakhov et al., 2015), their downstream effectors are often active on longer time scales and spread over longer distances. This has been studied mostly for the GTPases. After single synapse LTP induction, the GTPases Ras and Rac1 diffuse freely over approximately 10 μm within the dendrite and neighboring spines, while RhoA reaches ∼5 μm (Harvey et al., 2008; Murakoshi et al., 2011; Hedrick et al., 2016). Although Cdc42 is equally mobile as its family members, Cdc42 activation is contained within the spine head (Murakoshi et al., 2011).

Diffusion of these signaling molecules can reduce the threshold for LTP in neighboring spines and thereby mediate synaptic crosstalk. When Ras signaling is pharmacologically inhibited, crosstalk is reduced (Harvey et al., 2008). Similarly, interfering with the spread of Rac1 and RhoA activity out of suprathreshold spine significantly reduces crosstalk without affecting the growth of the suprathreshold spine (Hedrick et al., 2016). A subthreshold stimulus (using shorter glutamate pulses) does not activate Ras and only weakly activates Rac1 and RhoA. A suprathreshold stimulus on a spine nearby can elevate Ras, Rac1 and RhoA activation levels in the subthreshold spine above threshold. Cdc42 activation is similar after subthreshold and suprathreshold stimuli (Harvey et al., 2008; Hedrick et al., 2016).

During single synapse LTP, activation of PKC is almost completely restricted to the stimulated dendritic spine. However, when a nearby spine receives a subthreshold stimulus at the same time, PKC also gets activated in the subthreshold spine. PKC activation is triggered by fast and local calcium influx through NMDA receptors but is also sensitive to DAG production through TrkB-PLC signaling (Colgan et al., 2018). Because TrkB activation slowly spreads over a stretch of 10 μm (Harward et al., 2016), PKC may integrate the activation history of nearby spines (Colgan et al., 2018).

Both PKA and ERK activation spread over more than 10 μm of dendrite and invade nearby spines, with PKA showing a sharper spatial gradient and a more rapid decay than ERK (Tang and Yasuda, 2017). ERK can stay active for a long time and diffuse over long distances within the dendritic tree. LTP induction on at least 3 spines on two different branches within 30 min leads to sustained nuclear ERK activation that is likely mediated by diffusion of activated ERK from the stimulated spines. Nuclear ERK activation is dependent on mGluR activation and may require PKC to maintain ERK activation. In the nucleus, ERK likely activates transcription factors that are responsible for the late phase of LTP (Zhai et al., 2013).

While the crosstalk described above typically works on a time scale of a few minutes (Harvey and Svoboda, 2007), another form of crosstalk has been observed on longer time scales. In the study by Govindarajan et al. (2011), glutamate uncaging alone leads to spine growth that returns to baseline after 2 h, while combining glutamate uncaging with pharmacological PKA activation leads to protein synthesis-dependent LTP that lasts for at least 4 h. Interestingly, when a spine is exposed to glutamate uncaging alone before or after a neighboring spine is exposed to glutamate uncaging paired with PKA activation, both spines grow persistently for up to 4 h. This crosstalk works over a time range of tens of minutes (both pre and post) and tens of micrometers on the same dendritic branch, and depends on protein synthesis (Govindarajan et al., 2011).

Besides biochemical interactions, nearby spines will also interact electrically. Their postsynaptic potentials summate, often in non-linear ways (London and Häusser, 2005; Losonczy and Magee, 2006; Tran-Van-Minh et al., 2015). For instance, it was shown that when four spines on a distal dendritic segment are stimulated, calcium levels in individual spines are higher than when the spines are activated individually, and this is mediated by NMDA receptors. Simultaneous subthreshold stimulation at these spines (in the presence of magnesium and without depolarization) can overcome the LTP threshold and can induce functional LTP in these spines (Weber et al., 2016).

Together, these studies show that single synapse LTP is affected by the recent activity of nearby spines and mediated by many factors, such as local kinase activity and dendritic exchange of GTPases (Nishiyama and Yasuda, 2015; Yasuda, 2017). Crosstalk of LTP between neighboring spines along the same dendrite is particularly relevant *in vivo*, where synapses with similar properties or activity patterns often cluster together (Kleindienst et al., 2011; Makino and Malinow, 2011; Bloss et al., 2016, 2018; Wilson et al., 2016; Iacaruso et al., 2017).

### Plasticity and Crosstalk During Development

During development, the rules for synaptic plasticity and crosstalk are not the same as in mature neurons (Lohmann and Kessels, 2014). When uncaging at a single spine in young, developing neurons, calcium is less restricted in the spine head than in mature neurons, and calcium influx through NMDA channels can be boosted by calcium-induced calcium release (CICR) (Lee et al., 2016). Activating individual spines often leads to propagating calcium waves in the dendrite that are mediated by CICR from intracellular stores. However, propagating calcium waves after LTP induction have not been observed in more mature neurons, suggesting that the coupling between NMDA receptors and internal calcium stores is developmentally regulated. In young neurons, all spine growth depends on CICR, suggesting that calcium influx through NMDA receptors is not sufficient to induce LTP in young neurons. When a strong stimulus on one spine is paired with a weaker stimulus on a neighboring spine, this leads to sustained spine growth in both spines, and this crosstalk is also dependent on CICR (Figure 2B). In general, the high level of local crosstalk in young neurons suggests the clustered maturation of spines. Indeed, it was shown that mature synapses, which have high AMPA/NMDA ratios, tend to cluster together on dendrites of young neurons (Lee et al., 2016).

### Heterosynaptic Spine Shrinkage

When a small cluster of spines (at least four) is potentiated using glutamate uncaging, it can induce shrinkage and loss of AMPA receptors at an unstimulated spine close to that cluster (Figure 2C) (Oh et al., 2015). This heterosynaptic shrinkage is dependent on the calcium sensing protein calcineurin, mGluR and IP3 receptor signaling, but it is independent of the classical LTP protein CaMKII. When CaMKII is inhibited, spine growth at the stimulated spines is prevented but the unstimulated spine still shrinks. When calcineurin is inhibited, only growth of the stimulated spines remains. This shows that the spine is not shrinking because of competition for resources, but because it is actively being regulated (Oh et al., 2015).

Spine shrinkage can also be induced by combining single spine glutamate uncaging with activation of dendritic GABA_*A*_ receptors (Hayama et al., 2013). Neighboring spines within 15 μm also undergo shrinkage, and synaptic transmission is weakened. This type of spine shrinkage depends on NMDA receptor and calcineurin signaling but is independent of mGluR signaling. While shrinkage spreads over the dendrite, a neighboring spine receiving a potentiating stimulus can still overcome the shrinkage signals and grow (Hayama et al., 2013). Together, these studies show that parallel signaling pathways for spine growth and shrinkage exist within the dendrite.

### Interaction Between Excitatory and Inhibitory Synapses

Inhibitory synapses are important regulators of dendritic signals. They interact with excitatory synaptic inputs electrically, and they play an important role in regulating calcium dynamics in the dendrite (Higley, 2014). An individual inhibitory synapse can reduce the influx of calcium during a backpropagating action potential locally within the dendrite (Müllner et al., 2015) or even within a single spine (Chiu et al., 2013). Additionally, activation of metabotropic GABA_*B*_-receptors reduces NMDA receptor-mediated calcium influx in single activated spines (Chalifoux and Carter, 2010). Inhibitory synapses are therefore likely able to interfere with nearby single synapse LTP induction. It needs to be noted that most studies discussed in this review use MNI-glutamate as their caged compound, which has been shown to have strong antagonistic effects on GABA_*A*_ receptors (Fino et al., 2009; Matsuzaki et al., 2010; Ellis-Davies, 2019). In addition, the presence of TTX in experiments using magnesium-free induction protocols also abolishes spontaneous activity in inhibitory neurons. Inhibitory synaptic signaling might therefore be largely blocked in these studies, which may affect the induction and/or expression of single synapse LTP.

Vice versa, LTP at spines also affects nearby inhibitory synapses. Chemical and electrical LTP studies have shown that NMDA receptor activation affects gephyrin clusters and the surface expression of GABA_*A*_ receptors (Marsden et al., 2007; Petrini et al., 2014; Flores et al., 2015) and leads to strengthening of inhibitory inputs (Bourne and Harris, 2011; Chiu et al., 2018). Using glutamate uncaging, our lab has recently shown that activation of a cluster of excitatory synapses can trigger the growth of a new inhibitory presynaptic bouton onto the stimulated dendrite via NMDA receptors and a retrograde endocannabinoid signal (Figure 2D) (Hu et al., 2019). Such a local coordination mechanism between excitatory and inhibitory plasticity will be important in regulating a balance between excitatory and inhibitory synapses within a dendritic branch and ensuring local inhibitory control over an active excitatory cluster.

### Interaction With Homeostatic Plasticity

Homeostatic plasticity operates over long time scales to maintain neuronal network function (Turrigiano, 2012). Neurons can regulate their own excitability by different mechanisms, including synaptic scaling of AMPA receptors (Turrigiano et al., 1998). Although the intracellular signaling pathways underlying synaptic scaling are not entirely clear, it is not unlikely that they partially overlap, or even interfere, with single synapse LTP. Lee et al. (2010) performed single synapse LTP at spines with silent (e.g., tetanus toxin expressing) presynaptic terminals, which had undergone synaptic scaling. They showed that presynaptic silencing leads to a decrease in LTP threshold, such that a stimulus protocol that is normally subthreshold can induce spine growth and functional LTP at presynaptically silenced spines (Figure 2E). They did not observe a difference in LTP when a suprathreshold stimulus was used (Lee et al., 2010). This suggests that homeostatic plasticity at individual synapses can affect the threshold for inducing spine growth and LTP.

Similarly, a recent study by Hobbiss et al. (2018) shows that when action potentials are blocked in a hippocampal slice for 48 h using TTX, spines become bigger and stronger, indicative of synaptic scaling. Using glutamate uncaging, the authors showed that small spines that were exposed to TTX treatment grow more after an LTP stimulus than untreated spines of the same size. In addition, a weak stimulus that does not induce sustained spine growth under control conditions induces significant spine growth in the TTX condition. This suggests that homeostatic scaling enhances the capacity to undergo LTP (Hobbiss et al., 2018). However, in an earlier study by Soares et al. (2017), no differences were observed in uncaging-induced spine growth between control and TTX-treated conditions.

## Concluding Remarks

Since the first study reported LTP of a single dendritic spine using glutamate uncaging (Matsuzaki et al., 2004), several protocols have been used to induce single synapse LTP: magnesium-free protocols that do not require electrical access to the postsynaptic neuron or paired protocols attempting to resemble physiological activation of the postsynaptic neuron. The expression of LTP in a single synapse is measured by quantifying the increase in uEPSC amplitude and/or in spine size, which are highly correlated with one another. Thanks to tremendous technological advances, signaling pathways involved in single synapse LTP are studied with spectacularly high spatial and temporal resolution. Remarkably, these studies at the single synapse level revealed that synapses do not necessarily operate individually. Specific signaling proteins leave the spine head and penetrate the dendritic shaft and nearby spines, where they can reduce the threshold for LTP. This implies that the activation and plasticity history of the synapse itself, as well as the history of synapses in its direct dendritic vicinity, strongly influence its capacity to undergo plasticity.

While we gained significantly more insight into the mechanisms of LTP at the single synapse level over the past 15 years, several questions remain and new questions emerge. There is sufficient evidence to conclude that different induction protocols trigger different signaling pathways and lead to different “modes” or levels of LTP expression. Morphological changes (peak and plateau spine growth) and functional LTP (receptor insertion) are not always perfectly aligned and may be evoked via different molecular routes with different experimental induction protocols. It will be the next challenge to understand if these parallel LTP pathways matter under physiological circumstances.

Another major challenge for the field is to understand the systems that are in place to coordinate the multitude of synaptic inputs within the neuron. Synapses with similar properties tend to cluster together on the same dendritic branch (Kleindienst et al., 2011; Druckmann et al., 2014; Bloss et al., 2016, 2018; Wilson et al., 2016; Iacaruso et al., 2017). One could therefore argue that *in vivo*, LTP rarely happens at isolated synapses but perhaps more often at small clusters of co-active synapses. It is therefore important to understand how spines undergoing LTP can interact within dendrites. Several studies have now started to address the mechanisms behind different forms of crosstalk. Expanding these studies to larger clusters of synapses, and including excitatory as well as inhibitory synapses, will allow us to examine under which circumstances synapses cooperate and when they compete for resources.

Research has focused on LTP in single spines, but the current understanding of synaptic depression and shrinkage of dendritic spines is much more limited. Only two uncaging protocols are known to induce LTD in the stimulated spine, and one of those also requires GABA uncaging (Hayama et al., 2013; Oh et al., 2013). Spine shrinkage and synaptic depression are not regulated by the inverse of LTP pathways but involve specific signaling. It will be important for future research to further unravel the spatial and temporal profile of LTD-associated signals and to examine overlap and interaction with LTP pathways.

In recent years, caged GABA compounds became available for two-photon uncaging. While uncaging GABA has been used to identify and quantify the presence of GABA receptors (Kantevari et al., 2010; Kanemoto et al., 2011; Chiu et al., 2013; Villa et al., 2016; Kwon et al., 2018) and to induce nascent excitatory or inhibitory synapses in young neurons (Oh et al., 2016), two-photon GABA uncaging has yet to enter the realm of synaptic plasticity. It would be interesting to use GABA uncaging to assess changes in the strength of individual inhibitory synapses. Coordination between excitation and inhibition, which is crucial for the proper functioning of neurons, is regulated at the synaptic level (Liu, 2004; Chen et al., 2012, 2015; Bloss et al., 2016; Hu et al., 2019). We therefore expect that improving our understanding of the interaction of excitatory and inhibitory plasticity at the level of single synapses and dendrites, for example by combining two-photon uncaging of glutamate and GABA (Kantevari et al., 2010), will provide us with exciting new insights.

The dendritic branch can be considered the fundamental electrical and biochemical functional unit of the nervous system (Branco and Häusser, 2010; Govindarajan et al., 2011; Lovett-Barron et al., 2012). Single synapse LTP studies are revealing that the molecular signaling pathways underlying single synapse LTP are not limited to the stimulated spine, but kinases, GTPases and other regulators can travel and interact with proteins in the dendrite and neighboring synapses. The precise effect of synaptic activation depends therefore on the activation and plasticity history of the involved synapse as well as excitatory and inhibitory synapses in its direct vicinity. Therefore, synaptic plasticity should always be considered within the context of the local dendritic homeostasis.

## Author Contributions

Both authors wrote and revised the manuscript.

## Conflict of Interest

The authors declare that the research was conducted in the absence of any commercial or financial relationships that could be construed as a potential conflict of interest.
